# Prostaglandin production in brain endothelial cells during the initiation of fever

**DOI:** 10.1080/19420889.2023.2166237

**Published:** 2023-01-11

**Authors:** Anna Eskilsson, Kiseko Shionoya, Anders Blomqvist

**Affiliations:** Division of Neurobiology, Department of Biomedical and Clinical Sciences, Faculty of Medicine and Health Sciences, Linköping University, Linköping, Sweden

**Keywords:** Cyclooxygenase-2, microsomal prostaglandin E synthase-1, prostaglandin E_2_, lipopolysaccharide, brain vascular cells, myeloid cells

## Abstract

The initiation of fever has been a matter of controversy. Based on observations of little or no induction of prostaglandin synthesizing enzymes in the brain during the first phase of fever it was suggested that fever is initiated by prostaglandin released into the circulation from cells in the liver and lungs. Here we show in the mouse that prostaglandin synthesis is rapidly induced in the brain after immune challenge. These data are consistent with our recent findings in functional experiments that prostaglandin production in brain endothelial cells is both necessary and sufficient for the generation of all phases of fever.

Fever is a cardinal symptom of inflammatory and infectious diseases. Experimental models for the study of fever often make use of peripheral administration of bacterial wall lipopolysaccharides (LPS). Generally, LPS is injected intraperitoneally, which results in what appears to be a biphasic temperature response (Figure 1a) that follows the initial rapid hyperthermia, which, as shown by its appearance also in saline injected mice, is due to the handling stress associated with the injection procedure. However, as pointed out previously [1], when mice are injected with LPS intravenously, in a manner that avoids the handling stress, three febrile phases are seen, with a first phase appearing within 20–30 min (Figure 1b). It is likely that this initial febrile response is obscured by the handling stress-evoked hyperthermia that appears when LPS is given intraperitoneally.

**Figure 1.**
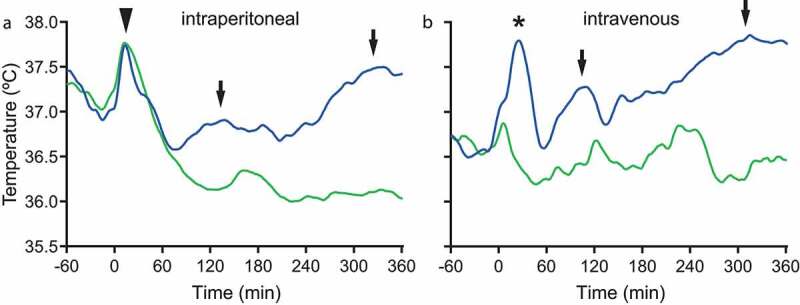
The febrile response to intraperitoneal and intravenous injection of lipopolysaccharide. a. Biphasic fever after intraperitoneal injection of 120 μg/kg LPS (blue trace). Arrowhead points at the handling stress induced hyperthermia and arrows at the two fever phases. b. Polyphasic fever after intravenous injection of 30 μg/kg LPS. Asterisk indicates 1^st^ phase of fever, and arrows point at the 2^nd^ and 3^rd^ phases, respectively. Green trace in a and b shows the response to saline injection. Injections were done at time point zero. The response to LPS and saline is compiled from a series of 13 and 20 mice, respectively, in a, and 9 and 6 mice, respectively, in b.

Fever is elicited by immune-induced synthesis of prostaglandin E_2_ (PGE_2_) and its binding to PGE_2_ EP_3_ receptors in the median preoptic nucleus of the hypothalamus [2,3], which in turn via descending pathways activate pre-sympathetic neurons resulting in peripheral vasoconstriction and thermogenesis. While there has been accumulating evidence that PGE_2_ production in brain vascular cells is involved in the second and third phases of fever (for refs., see [4]), data have been contradictory as to how fever is initiated. Based on observations in rats indicating that during the first phase of fever there was little or no induced transcription or protein expression of the PGE_2_ synthesizing enzymes cyclooxygenase-2 (Cox-2) and microsomal prostaglandin E synthase-1 (mPGES-1) in the brain whereas strong such induction was seen in liver and lung [5], it was suggested that the first phase of fever was elicited by PGE_2_ synthesized and released into the circulation by peripheral macrophages [6,7]. This idea was further supported by the finding that fever evoked by intravenous injection of PGE_2_ was attenuated by concomitant injection of PGE_2_ neutralizing antibodies [6]. However, using chimeric mice, generated by whole body irradiation followed by bone-marrow transplantation, we demonstrated that while mice with mPGES-1 expressed by hematopoietic cells (including macrophages), but not by non-hematopoietic cells, showed induced levels of PGE_2_ metabolites in blood, such mice did not display fever. Conversely, mice with extensive replacement of their hematopoietic cells with mPGES-1 KO cells still displayed a febrile response, including the first phase of fever [8]. Recently, we reported [9] that mice with deletion of Cox-2 in myeloid cells displayed a normal febrile response to i.v. injection of LPS. The body temperature recordings, not previously shown, are displayed in Figure 2. The findings indicate that prostaglandin production by macrophages is not involved in any of the febrile phases, at least not in the mouse.

**Figure 2.**
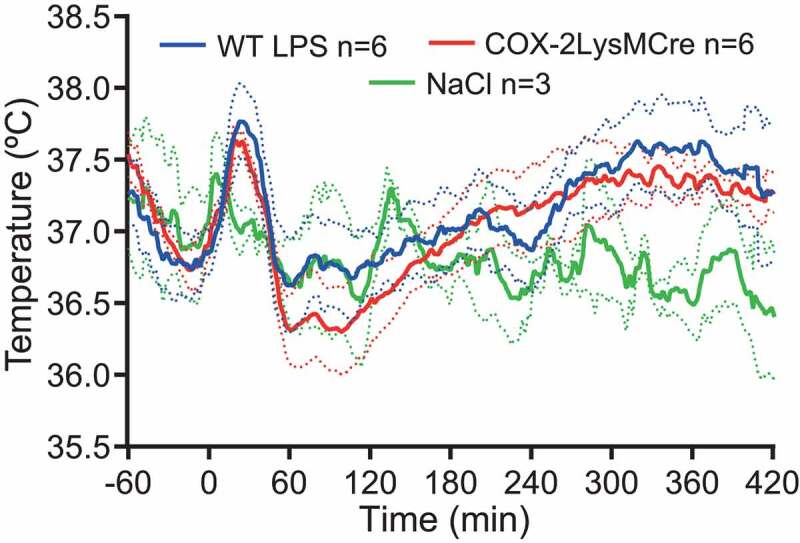
Fever in mice with cyclooxygenase-2 deletion in myeloid cells. WT mice and COX-2LysMCre mice show similar febrile responses to i.v. injection of 30 μg/kg LPS.

We recently also demonstrated, using mice with specific deletion and expression, respectively, of PGE_2_ synthesizing enzymes in brain endothelial cells that PGE_2_ production by these cells is both necessary and sufficient for the generation of LPS-induced fever, including its first phase [9]. We here report some additional data on induced prostaglandin synthesis in the brain that are consistent with the results of the functional studies while differing from those previously reported in rats.

Figure 3a shows the results of a qPCR analysis of the expression of Cox-1, Cox-2, and mPGES-1 in the hypothalamus of mice that had been immune challenged with i.v. injection of LPS (30 µg/kg) and killed 30 min later. There was strong induction of Cox-2 mRNA, which was associated with induced Cox-2 immunoreactivity in brain vessels (Figure 3c-e). There was no induction of the constitutive Cox isoform, Cox-1, as would be expected, but neither of the PGE_2_ terminal isomerase mPGES-1 (Figure 3a). It should be noted however that whereas there are low constitutive levels of mPGES-1 in the brain of rats [10], mPGES-1 is constitutively expressed in several cell types in the brain of mice, including brain endothelial cells [11]. While analysis of brain endothelial cells from mice obtained by FACS demonstrates severalfold induction of the mPGES-1 gene in these cells in response to peripheral immune challenge [12], qPCR analysis of whole brain tissue shows a modest two-fold induction of mPGES-1 at the height of the LPS-induced inflammatory response [13]. It is hence possible that some induction of mPGES-1 was obscured in the present qPCR analysis by the prominent constitutive mPGES-1 expression. However, it is also possible that its constitutive expression is sufficient for the manifestation of fever since Cox-2 is generally regarded as the rate limiting enzyme for the prostanoid synthesis [14]. Being in line with rapid induction of Cox-2 in the brain, we found increased levels of PGE_2_ in the cerebrospinal fluid at 30 min after the immune challenge (Figure 3b); however, it should be recognized that the latter observation shows correlation and not causality.

**Figure 3.**
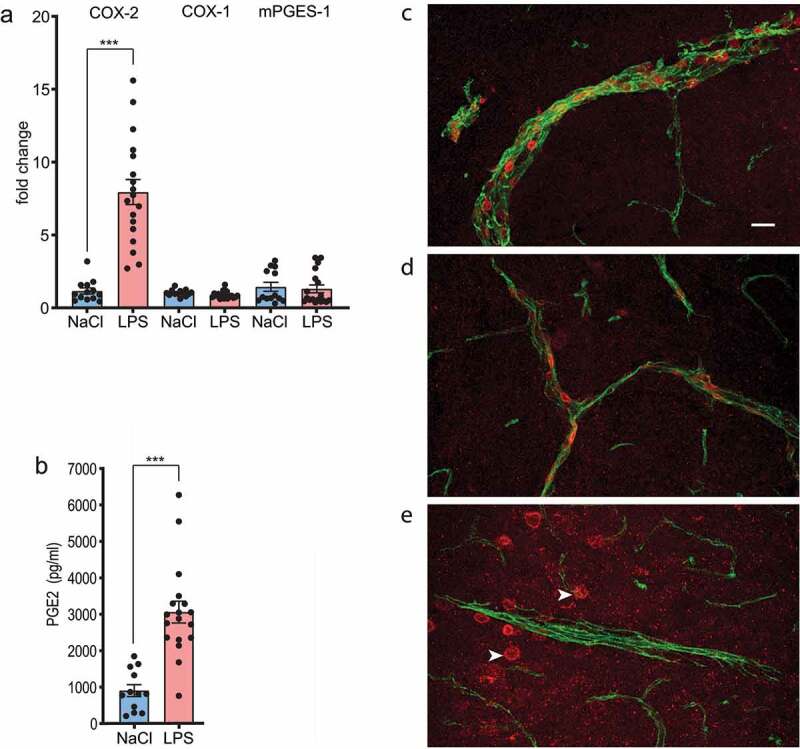
Prostaglandin synthesis in the brain during the initial febrile response to i.v. injection of LPS. a. Induced expression of Cox-2 mRNA, but not of Cox-1 or mPGES-1 mRNA in the hypothalamus 30 min after injection. b. PGE_2_ levels in the cerebrospinal fluid 30 min after injection. Error bars = SEM. *** *P* < 0.001 (unpaired *t*-test). c-e. Confocal micrographs showing induced Cox-2 immunoreactivity (red) in large (c) and small (d) blood vessels, stained for the endothelial cell marker CD31 (green), 30 min after LPS injection. After saline injection (e), there was no Cox-2 in the blood vessels, however, staining of cortical neurons (arrowheads) was seen, demonstrating method accuracy. Scale bar = 20 μm.

The data shown here hence provide supporting evidence for our demonstration that prostaglandin production in brain endothelial cells is the source fever, including its first phase. While previously strong induction of Cox-2 mRNA and Cox-2 protein consistently has been shown in brain endothelial cells during the later phases of fever [8,13,15], we here demonstrate that also during the first phase of fever Cox-2 mRNA is induced in the brain and Cox-2 protein expressed in brain endothelial cells, and that both phenomena at this early time-point are associated with increased levels of brain PGE_2_. We can hence conclude that at least in the mouse the available data are consistent with our finding that brain endothelial cells are the critical components for the generation of all phases of fever. What now needs to be explored is the nature of the stimulus that rapidly activates the brain endothelial cells. While it has been demonstrated that the LPS-induced febrile response during the later febrile phases is dependent on the presence of receptors for IL-1 and IL-6 on brain endothelial cells [16,17], it is not known if the initial febrile response also is elicited by cytokine signaling in these cells or whether it comes into being through direct activation of the endothelial cells by LPS through binding to endothelial TLR4 [12].

## Materials and methods

### Animals

Adult wild-type mice of both sexes of the C57BL/6J strain were obtained from Janvier labs. Mice with selective deletion of Cox-2 in myeloid cells were generated by crossing mice possessing loxP sites flanking exons 4–5 of the Ptgs2 gene [18] with mice expressing Cre recombinase under the LysM promoter [19]. The mice were housed under constant ambient temperature (21°C), on a 12 h/12 h light/dark cycle (lights on at 7 A.M.) with food and water available ad libitum. All animal experiments were approved by the Animal Care and Use Committee at Linköping University (#1854/2018).

### Surgery

Mice were anesthetized with ketamine (70 mg/kg) and dexmedetomidine (0.4 mg/kg) and implanted i.p. with a transponder that records core body temperature (E-Mitter; Starr Life Sciences). Mice intended for intravenous injections were during the same session, or as a single procedure, provided with an indwelling jugular catheter that was exteriorized at the back of the neck and connected to a swivel system (CMA Microdialysis) on the top of the cage, permitting injections without handling the mice [8]. Following surgery, the mice were kept at an ambient temperature of 29°C, providing near-thermoneutral conditions [20]. Intravenous injections (30 μg/kg LPS or saline) were given 4 days after surgery, whereas intraperitoneal injections (120 μg/kg LPS or saline) were given about 7 days after surgery.

### Immunohistochemistry

The animals were perfused transcardially with a phosphate-buffered (0.1 M; pH 7.4) paraformaldehyde solution (4%). The brain was removed and postfixed for 3 h, then cryoprotected with 30% sucrose in PBS, and cut at 30 μm on a freezing microtome. The immunohistochemical procedures were performed according to standardized protocols [8]. Primary antibodies were polyclonal rabbit anti-Cox-2 (Santa Cruz, M-19, sc-1747-R; diluted 1:500), detected with Alexa Fluor 555 donkey anti-rabbit, 1:1000 (Life Technologies, Cat. no. A31572), and purified rat monoclonal anti-CD31 (AbD Serotec, MCA2388GA; 1:1000), detected with Alexa Fluor 488 donkey anti-rat (Life Technologies, Cat. no. A21208; 1:500).

### Immunoassay and real-time PCR

The mice were killed by asphyxiation with CO_2_ and placed in a stereotaxic frame. The atlanto-occipital membrane was exposed, and cerebrospinal fluid was withdrawn from the cisterna magna using a Hamilton syringe mounted on a micromanipulator and immediately frozen. A hypothalamic block was then dissected and placed in RNAlater stabilization reagent (Qiagen) and stored at −70°C until analysis.

The concentration of PGE_2_ in the cerebrospinal fluid (diluted 1:100) was determined using a High Sensitivity Prostaglandin E2 Enzyme Immunoassay Kit (Assay Designs). The values were calculated using a standard curve ranging from 7.81 to 1000 pg/ml (*R*^2^ = 1).

RNA was extracted from the hypothalamus with RNeasy Plus Universal Kit (Qiagen), and reverse transcription was done with High-Capacity cDNA Reverse Transcription Kit (Applied Biosystems); qPCR was performed using Gene Expression Master Mix (Applied Biosystems) on a 96-well plate (7500 Fast Real-Time PCR System Software; Applied Biosystems). Mm00477214_m1 (Cox-1), Mm00478374_m1 (Cox-2), Mm00452105_m1 (mPGES-1), and Mm99999915_g1 (GAPDH) TaqMan assays (Applied Biosystems) were used.
